# Complete genome sequence of a carbon tetrachloride-degrading bacterium, *Pseudomonas* sp. strain Stari2

**DOI:** 10.1128/mra.00958-24

**Published:** 2025-01-29

**Authors:** Leonardo Stari, Thiti Jittayasotorn, Chihiro Inoue, Mei-Fang Chien

**Affiliations:** 1 Graduate School of Environmental Studies, Tohoku University, Sendai, Japan; 2 Graduate School of Life Science, Tohoku University, Sendai, Japan; Loyola University Chicago, Chicago, Illinois, USA

**Keywords:** *Pseudomonas*, carbon tetrachloride

## Abstract

This article reports on the complete genome of *Pseudomonas* sp. strain Stari2, which was shown to have the ability to degrade carbon tetrachloride (CCl_4_) in aerobic conditions. A single circular sequence of 6,310,573 bp, a GC content of 60.3%, and 5,680 coding sequences were obtained. *In silico* analysis revealed an average nucleotide identity of 94.06% to its closest species.

## ANNOUNCEMENT

CCl_4_ (CT) is a priority environmental contaminant due to its high toxicity, which poses significant risks to human health and ecosystems through the generation of free radicals via oxidative pathways (1). While CT biodegradation has been extensively studied under anaerobic conditions primarily through cometabolic reduction (2–4), aerobic degradation remains less explored. Our previous work isolated a bacterium capable of aerobic CT degradation from a microbial consortium collected in Iwate Prefecture, Japan (5).

Isolated bacteria were grown in brain heart infusion medium with 5 mM CT at 30°C for 48 h, and high-molecular weight (HMW) DNA was extracted using the Promega Wizard HMW DNA Extraction Kit following the manufacturer’s protocol. DNA was sequenced by Bioengineering Lab Co., Ltd. (Kanagawa, Japan) using two platforms: DNBSEQ (MGI, Shenzhen, China) for short reads and GridION (Oxford Nanopore Technologies, Oxford, UK) for long reads. Default parameters were used for all software, unless otherwise specified.

For short read sequencing, DNA concentration was measured using Synergy LX and QuantiFluor, and quality was confirmed via electrophoresis using the 5200 Fragment Analyzer System with the Agilent HS Genomic DNA 50 kb Kit. Library preparation utilized the MGIEasy FS DNA Library Prep Set and the MGIEasy DNA Adapters-96 Kit. Sequencing was performed under 2 × 200 bp conditions on the DNBSEQ-G400 platform. For long-read sequencing, the Ligation Sequencing Kit from Oxford Nanopore Technologies was used, and sequencing was performed on the GridION platform with an R9.4.1 flow cell. Guppy (Ver. 4.0.11) was used for real-time base calling and barcode sorting.

Adapter sequences from short reads were removed using Cutadapt (Ver. 2.7) (6), and read sampling was performed with SeqKit (Ver. 0.11.0) (7). Low-quality bases were removed using Sickle (version 1.33)(8). Long read adapter sequences were removed with Porechop (Ver. 0.2.3), and reads shorter than 1,000 bases were discarded using Filtlong (Ver. 0.2.0). Hybrid assembly of high-quality short and long reads was conducted using Unicycler (Ver. 0.4.7) (9). Assembly results were confirmed with Bandage (Ver. 0.8.1) (10), and genome data integrity was checked with CheckM (Ver. 1.1.2) (11). Gene prediction was carried out using PGAP (Ver. 6.1) (12).

The DNBSEQ and GridION sequencing generated 5,998,880 and 223,095 high-quality paired reads (N50 3,836 bp), respectively (paired raw reads 20,716,044 and 1,113,507 respectively). The resulting assembly consists of one circular chromosome with an N50 value of 6,310,573 bp, 60.3% GC content, and a coverage of 875×. Average nucleotide identity analysis (13) showed 94.06% similarity to *Pseudomonas kribbensis* strain 46-2 (type strain, GenBank accession CP029608 [14]), which is lower than the 95% threshold used to distinguish species (15).

*In silico* analysis of this complete genome using Prokka, BlastKoala, GhostKoala, KofamKoala, and EggNogMapper (16–19) indicated that strain Stari2 has complete pathways for carbon metabolism, partial pathways for nitrogen metabolism, and complete pathways for sulfur metabolism and cofactor biosynthesis. Fifteen CDSs potentially involved in dehalogenation were identified, including four associated with aerobic dehalogenation (Table 1). While enzymes for chloroalkane and chloroalkene degradation were detected, none were specific to carbon tetrachloride (CCl₄).

**TABLE 1:** CDSs potentially involved in dehalogenation in strain Stari2

Locus tag	Putative homolog	Substrate	Reference
NH234_02775	*dhaA*	R-X	(20)
NH234_21655	*l-dex*	C_2_HO_2_RX	(21)
NH234_03495	*ctrA*	CHCl_3_, 1,1,1-TCA	(22)
NH234_20080	*cfrA*	CHCl_3_	(23)
NH234_09950	*hadL*	C_2_HO_2_RX	(24)
NH234_00545	*dcmA*	CH_2_Cl_2_	(25)
NH234_03490	*pceA*	PCE, hexachloroethane	(26)
NH234_15595	*tceA*	TCE, cis-DCE, 1,1-DCE	(27)
NH234_14700	*cyp108*	CT and many others	(28)
NH234_07415	*tcbC*	1,2-Dichlorobenzene	(29)
NH234_14015	*cmuA*	Chloromethane	(30)
NH234_19170	*vcrA*	Chloroethene	(31)
NH234_20830	*dehH1*	Chloroacetate	(32)
NH234_22340	*trmA*	CHCl_3_	(33)
NH234_00830	*mmoC*	R-X	(34)

## Data Availability

The raw reads of *Pseudomonas* sp. strain Stari2 obtained from DNBSEQ and NANOPORE are available at SRA under accession numbers MN272363.1, SRX25506002, and SRX25506003. The full genome sequence can be accessed through accession number CP099760.

## References

[1] Knox RC, Canter LW. 1996. Prioritization of ground water contaminants and sources. Water Air Soil Pollut 88:205–226. doi:10.1007/BF00294102

[2] Lewis TA, Paszczynski A, Gordon-Wylie SW, Jeedigunta S, Lee C-H, Crawford RL. 2001. Carbon tetrachloride dechlorination by the bacterial transition metal chelator pyridine-2,6-bis(thiocarboxylic acid). Environ Sci Technol 35:552–559. doi:10.1021/es001419s 11351728

[3] Penny C, Gruffaz C, Nadalig T, Cauchie H-M, Vuilleumier S, Bringel F. 2015. Tetrachloromethane-degrading bacterial enrichment cultures and isolates from a contaminated aquifer. Microorganisms 3:327–343. doi:10.3390/microorganisms3030327 27682092 PMC5023247

[4] Kwon K, Shim H, Bae W, Oh J, Bae J. 2016. Simultaneous biodegradation of carbon tetrachloride and trichloroethylene in a coupled anaerobic/aerobic biobarrier. J Hazard Mater 313:60–67. doi:10.1016/j.jhazmat.2016.03.057 27054665

[5] Stari L, Tusher TR, Inoue C, Chien M-F. 2023. A microbial consortium led by A novel Pseudomonas species enables degradation of carbon tetrachloride under aerobic conditions. Chemosphere 319:137988. doi:10.1016/j.chemosphere.2023.137988 36724852

[6] Martin M. 2011. Cutadapt removes adapter sequences from high-throughput sequencing reads. EMBnet J 17:10. doi:10.14806/ej.17.1.200

[7] Shen W, Le S, Li Y, Hu F. 2016. SeqKit: a cross-platform and ultrafast toolkit for FASTA/Q file manipulation. PLoS One 11:e0163962. doi:10.1371/journal.pone.0163962 27706213 PMC5051824

[8] Joshi NA, Fass J. 2011 Sickle: a sliding-window, adaptive, quality-based trimming tool for FastQ files

[9] Wick RR, Judd LM, Gorrie CL, Holt KE. 2017. Unicycler: resolving bacterial genome assemblies from short and long sequencing reads. PLoS Comput Biol 13:e1005595. doi:10.1371/journal.pcbi.1005595 28594827 PMC5481147

[10] Wick RR, Schultz MB, Zobel J, Holt KE. 2015. Bandage: interactive visualization of de novo genome assemblies. Bioinformatics 31:3350–3352. doi:10.1093/bioinformatics/btv383 26099265 PMC4595904

[11] Parks DH, Imelfort M, Skennerton CT, Hugenholtz P, Tyson GW. 2015. CheckM: assessing the quality of microbial genomes recovered from isolates, single cells, and metagenomes. Genome Res 25:1043–1055. doi:10.1101/gr.186072.114 25977477 PMC4484387

[12] Li W, O’Neill KR, Haft DH, Dicuccio M, Chetvernin V, Badretdin A, Coulouris G, Chitsaz F, Derbyshire MK, Durkin AS, Gonzales NR, Gwadz M, Lanczycki CJ, Song JS, Thanki N, Wang J, Yamashita RA, Yang M, Zheng C, Marchler-Bauer A, Thibaud-Nissen F. 2020. RefSeq: expanding the prokaryotic genome annotation pipeline reach with protein family model curation. Nucleic Acids Res:D1020. doi:10.1093/nar/gkaa1105 PMC7779008 33270901

[13] Ha SM, Kim CK, Roh J, Byun JH, Yang SJ, Choi SB, Chun J, Yong D. 2019. Application of the whole genome-based bacterial identification system, truebac id, using clinical isolates that were not identified with three matrix-assisted laser desorption/ionization time-of-flight mass spectrometry (MALDI-TOF MS) Systems. Ann Lab Med 39:530–536. doi:10.3343/alm.2019.39.6.530 31240880 PMC6660342

[14] Chang D-H, Rhee M-S, Kim J-S, Lee Y, Park MY, Kim H, Lee S-G, Kim B-C. 2016. Pseudomonas kribbensis sp. nov., isolated from garden soils in Daejeon, Korea. Antonie Van Leeuwenhoek 109:1433–1446. doi:10.1007/s10482-016-0743-0 27460204

[15] Jain C, Rodriguez-R LM, Phillippy AM, Konstantinidis KT, Aluru S. 2018. High throughput ANI analysis of 90K prokaryotic genomes reveals clear species boundaries. Nat Commun 9:5114. doi:10.1038/s41467-018-07641-9 30504855 PMC6269478

[16] Aramaki T, Blanc-Mathieu R, Endo H, Ohkubo K, Kanehisa M, Goto S, Ogata H. 2020. KofamKOALA: KEGG Ortholog assignment based on profile HMM and adaptive score threshold. Bioinformatics 36:2251–2252. doi:10.1093/bioinformatics/btz859 31742321 PMC7141845

[17] Cantalapiedra CP, Hernández-Plaza A, Letunic I, Bork P, Huerta-Cepas J. 2021. eggNOG-mapper v2: functional annotation, orthology assignments, and domain prediction at the metagenomic scale. Mol Biol Evol 38:5825–5829. doi:10.1093/molbev/msab293 34597405 PMC8662613

[18] Kanehisa M, Goto S. 2000. KEGG: kyoto encyclopedia of genes and genomes. Nucleic Acids Res 28:27–30. doi:10.1093/nar/28.1.27 10592173 PMC102409

[19] Kanehisa M, Sato Y, Morishima K. 2016. BlastKOALA and GhostKOALA: KEGG tools for functional characterization of genome and metagenome sequences. J Mol Biol 428:726–731. doi:10.1016/j.jmb.2015.11.006 26585406

[20] Cole ST, Brosch R, Parkhill J, Garnier T, Churcher C, Harris D, Gordon SV, Eiglmeier K, Gas S, Barry CE 3rd, et al.. 1998. Deciphering the biology of Mycobacterium tuberculosis from the complete genome sequence. Nature New Biol 393:537–544. doi:10.1038/31159 9634230

[21] Köhler R, Brokamp A, Schwarze R, Reiting RH, Schmidt FRJ. 1998. Characteristics and DNA-sequence of a cryptic haloalkanoic acid dehalogenase from Agrobacterium tumefaciens RS5. Curr Microbiol 36:96–101. doi:10.1007/s002849900286 9425247

[22] Ding C, Zhao S, He J. 2014. A Desulfitobacterium sp. strain PR reductively dechlorinates both 1,1,1-trichloroethane and chloroform. Environ Microbiol 16:3387–3397. doi:10.1111/1462-2920.12387 24428759

[23] Tang S, Gong Y, Edwards EA. 2012. Semi-automatic in silico gap closure enabled de novo assembly of two Dehalobacter genomes from metagenomic data. PLoS One 7:e52038. doi:10.1371/journal.pone.0052038 23284863 PMC3528712

[24] Nardi-Dei V, Kurihara T, Okamura T, Liu JQ, Koshikawa H, Ozaki H, Terashima Y, Esaki N, Soda K. 1994. Comparative studies of genes encoding thermostable L-2-halo acid dehalogenase from Pseudomonas sp. strain YL, other dehalogenases, and two related hypothetical proteins from Escherichia coli. Appl Environ Microbiol 60:3375–3380. doi:10.1128/aem.60.9.3375-3380.1994 7944368 PMC201812

[25] Vuilleumier S, Chistoserdova L, Lee M-C, Bringel F, Lajus A, Zhou Y, Gourion B, Barbe V, Chang J, Cruveiller S, et al.. 2009. Methylobacterium genome sequences:: a reference blueprint to investigate microbial metabolism of C1 compounds from natural and industrial sources. PLoS ONE 4:e5584. doi:10.1371/journal.pone.0005584 19440302 PMC2680597

[26] Maillard J, Schumacher W, Vazquez F, Regeard C, Hagen WR, Holliger C. 2003. Characterization of the corrinoid iron-sulfur protein tetrachloroethene reductive dehalogenase of Dehalobacter restrictus. Appl Environ Microbiol 69:4628–4638. doi:10.1128/AEM.69.8.4628-4638.2003 12902251 PMC169082

[27] West KA, Lee PKH, Johnson DR, Zinder SH, Alvarez-Cohen L. 2013. Global gene expression of Dehalococcoides within a robust dynamic TCE-dechlorinating community under conditions of periodic substrate supply. Biotechnol Bioeng 110:1333–1341. doi:10.1002/bit.24819 23280440

[28] World Health Organization. 1999. Edited by J. de Fouw. Carbon tetrachloride. Environmental Health Criteria 208. World Health Organization, Geneva PP - Geneva. https://iris.who.int/bitstream/handle/10665/42133/WHO_EHC_208.pdf?sequence=1&isAllowed=y.

[29] van der Meer JR, Eggen RI, Zehnder AJ, de Vos WM. 1991. Sequence analysis of the Pseudomonas sp. strain P51 tcb gene cluster, which encodes metabolism of chlorinated catechols: evidence for specialization of catechol 1,2-dioxygenases for chlorinated substrates. J Bacteriol 173:2425–2434. doi:10.1128/jb.173.8.2425-2434.1991 2013566 PMC207804

[30] Richter N, Zepeck F, Kroutil W. 2015. Cobalamin-dependent enzymatic O-, N-, and S-demethylation. Trends Biotechnol 33:371–373. doi:10.1016/j.tibtech.2015.03.011 25887333

[31] Dolinová I, Štrojsová M, Černík M, Němeček J, Macháčková J, Ševců A. 2017. Microbial degradation of chloroethenes: a review. Environ Sci Pollut Res Int 24:13262–13283. doi:10.1007/s11356-017-8867-y 28378313

[32] Fetzner S. 1998. MINI-REVIEW Bacterial dehalogenation. Appl Microbiol Biotechnol 50. doi:10.1007/s002530051346 9891928

[33] Jugder B-E, Bohl S, Lebhar H, Healey RD, Manefield M, Marquis CP, Lee M. 2017. A bacterial chloroform reductive dehalogenase: purification and biochemical characterization. Microb Biotechnol 10:1640–1648. doi:10.1111/1751-7915.12745 28631300 PMC5658581

[34] Han JI, Semrau JD. 2000. Chloromethane stimulates growth of Methylomicrobium album BG8 on methanol . FEMS Microbiol Lett 187:77–81. doi:10.1111/j.1574-6968.2000.tb09140.x 10828404

